# Modelling of hydrogen sulfide fate and emissions in extended aeration sewage treatment plant using TOXCHEM simulations

**DOI:** 10.1038/s41598-020-79395-8

**Published:** 2020-12-17

**Authors:** Haider M. Zwain, Basim K. Nile, Ahmed M. Faris, Mohammadtaghi Vakili, Irvan Dahlan

**Affiliations:** 1 College of Water Resources Engineering, Al-Qasim Green University, Al-Qasim, Babylon 51013 Iraq; 2 Department of Civil and Architectural Engineering, College of Engineering, Sultan Qaboos University, Al Khoudh, P.O. Box 33, 123 Muscat, Oman; 3 College of Engineering, University of Kerbala, Kerbala, 56100 Iraq; 4 School of Civil Engineering, Iran University of Science and Technology, 1684613114 Narmak, Tehran Iran; 5 Kerbala Sewerage Directorate, Kerbala, 56001 Iraq; 6 Green Intelligence Environmental School, Yangtze Normal University, Chongqing, 408100 China; 7 School of Chemical Engineering, Universiti Sains Malaysia, Engineering Campus, Seri Ampangan, 14300 Nibong Tebal, Penang Malaysia; 8 Solid Waste Management Cluster, Science and Engineering Research Centre, Universiti Sains Malaysia, Engineering Campus, Seri Ampangan, 14300 Nibong Tebal, Penang Malaysia

**Keywords:** Civil engineering, Environmental monitoring, Pollution remediation

## Abstract

Odors due to the emission of hydrogen sulfide (H_2_S) have been a concern in the sewage treatment plants over the last decades. H_2_S fate and emissions from extended aeration activated sludge (EAAS) system in Muharram Aisha-sewage treatment plant (MA-STP) were studied using TOXCHEM model. Sensitivity analysis at different aeration flowrate, H_2_S loading rate, wastewater pH, wastewater temperature and wind speed were studied. The predicted data were validated against actual results, where all the data were validated within the limits, and the statistical evaluation of normalized mean square error (NMSE), geometric variance (VG), and correlation coefficient (R) were close to the ideal fit. The results showed that the major processes occurring in the system were degradation and emission. During summer (27 °C) and winter (12 °C), about 25 and 23%, 1 and 2%, 2 and 2%, and 72 and 73% were fated as emitted to air, discharged with effluent, sorbed to sludge, and biodegraded, respectively. At summer and winter, the total emitted concentrations of H_2_S were 6.403 and 5.614 ppm, respectively. The sensitivity results indicated that aeration flowrate, H_2_S loading rate and wastewater pH highly influenced the emission and degradation of H_2_S processes compared to wastewater temperature and wind speed. To conclude, TOXCHEM model successfully predicted the H_2_S fate and emissions in EAAS system.

## Introduction

Odors emission from sewage treatment plants (STPs) are longstanding environmental issue that receives ongoing attention as a result of urbanization and population expansion. The emission of odors from STPs causes unpleasant nuisances for plant workers and people nearby. Odors cause several health effects such as headache, nausea, and respiratory-related issues. Some odors can be toxic and cause adverse health impacts such as death. Furthermore, odor has negative social economic effects by reducing the price of properties and prospects for tourism due to esthetic nuisance^1^.

Apart from that, odor problems close to STPs were associated with hydrogen sulfide (H_2_S) emission as a major source for annoying odors even at very low concentrations. H_2_S is colorless, flammable, associated with a rotten egg smell and very toxic gas. H_2_S is generated from the combination of decomposition of organic sulfur from feces and reduction of inorganic sulfur compounds from the sulfate ion (SO_4_^2−^) by bacteria and archaea under anaerobic conditions^2^. H_2_S has very low odor threshold limit, however its malodor can even be noticed below 1 ppm, whereas human’s odor threshold between 0.0005 and 1.5 ppm. Long human’s exposure (8 h) to concentrations ranges from 2 to 5 ppm causes headache, nausea and tearing of eyes, while concentration of 50 ppm causes respiratory tract irritation. Single exposure to 500 ppm results in sudden unconsciousness and death if the levels are over 1000 ppm^3^.

Despite that, there is a high lack of clear legal acts and guidelines regulating H_2_S emission and dispersion. Hence, a proper control of it is important to reduce nuisances experienced by the exposed populations. The direct way of controlling human exposure to odor is by avoiding the discharge of odor from the origin. Baawain et al*.*^4^ reported that specific odor exposure can primarily be quantified by the integral results of sources of emission, dispersion route, and characteristics of receptor. Different methods (i.e. models, surveys and chamber monitoring) have been used to study odor nuisance to estimate the degree of odors emission from STP^1,2,5,6^. The management of H_2_S can be assisted by mathematical models to understand its fate and emission. The mechanisms of pollutants removal in these models are the degradation and volatilization from different processes. Such mechanisms depends on biological reactions and mass transfer in the liquid and gas phases^7^.

Accordingly, TOXCHEM model is an efficient tool for the prediction of volatile organic compounds (VOCs) fate and hazardous air pollutants (HAPs) emission within/from wastewater treatments plants (WWTPs). As an alternative to Water9 software, TOXCHEM was first developed in early 1990s by US Environmental Protection Agency to overcome limitations of Water9. It is based on mass balance of several compounds in WWTPs for each operation unit, taking into account many physical, chemical, and biological processes such as sorption, stripping, volatilization and biodegradation. It is mainly used for VOC air emissions estimates from wastewater collection systems, WWTPs, and disposal facilities. This also include the reduction of air emission by planning, designing and optimization of process projects. In addition, it can also be implemented to predict the loads/concentrations of contaminants in the water effluent, and residual solids streams^8^.

Karbala state is geologically characterized by gypsum soil and high levels of groundwater, especially in the district where the plant is located. Hence, the groundwater contains very high concentrations of sulfide (SO_4_^2−^) compounds that infiltrate into the sewer system, leading to increased SO_4_^2−^ concentrations in sewage, in addition to several other wastewater sources containing SO_4_^2−^. In that sewer systems, sewage are going through oxygen depletion, variable flow rate and velocity, and long retention time that consequently lead to the decomposition of SO_4_^2−^ to H_2_S gas dissolved in wastewater. As a results, Muharram Aisha sewage treatment plant (MA-STP) is receiving high concentrations of H_2_S that result in the emission of sever odors in the area, which casing problems to the workers and people in the surrounding area. Studies on the modelling of odors exposure associated with H_2_S from STPs’ are very limited. Therefore, the study aims to model the H_2_S fate at different treatment units of MA-STP and emission from these units to the atmosphere, during summer and winter, using TOXCHEM V4.1 simulations. In addition, sensitivity analysis is conducted to understand the effect of variation in aeration flowrate, H_2_S loading rate, wastewater pH level, wastewater temperature and wind speed on the fate and emission of H_2_S.

## Materials and methods

### Site location and description

Muhhram Aisha sewage treatment plant (MA-STP) is located in Al-Hindiya District, at about 20 km from the center of the Karbala, and nearly 110 km to the south of Baghdad, the capital of Iraq.

**Figure 1:**
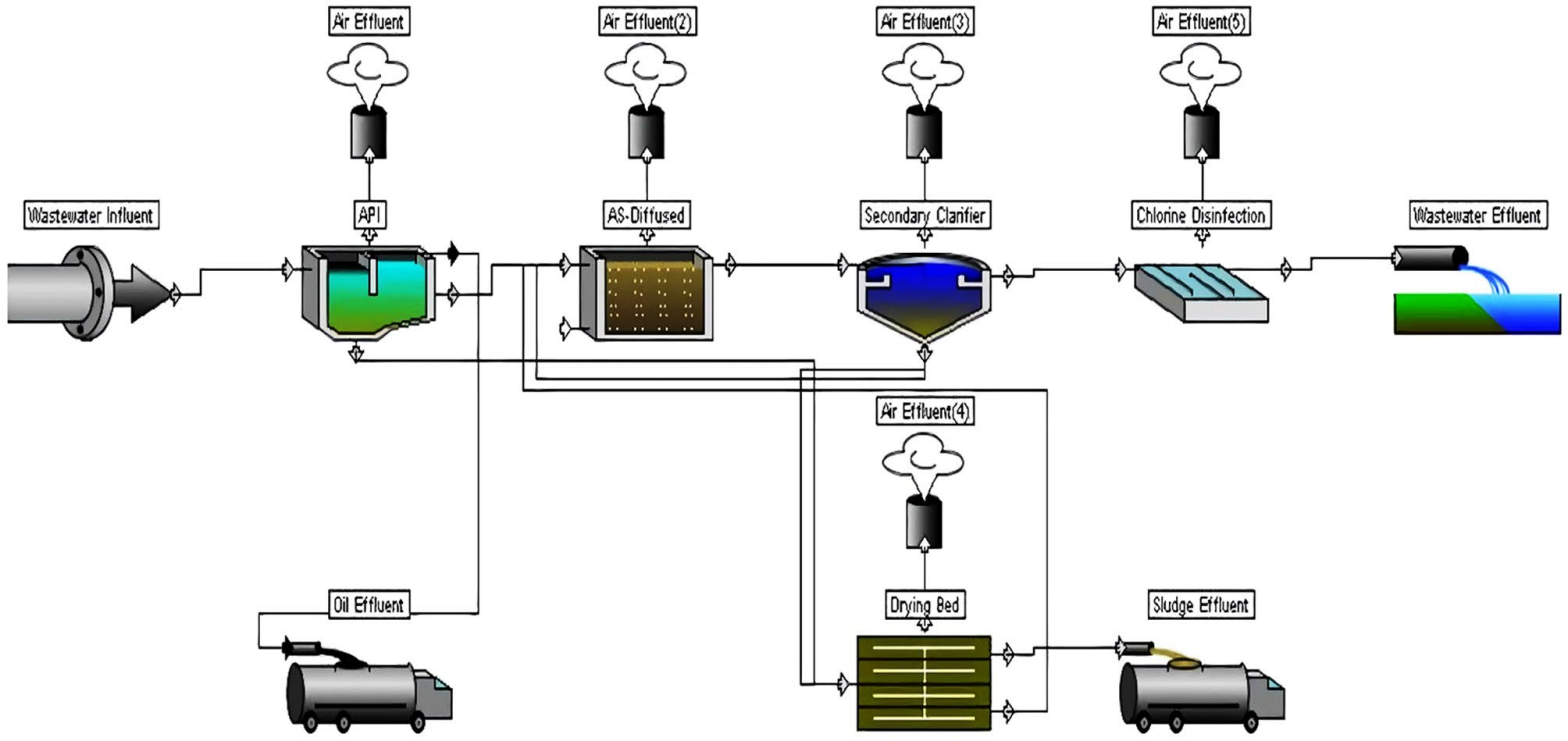
Schematic diagram of EEAS system in MA-STP.

The geographical coordinates of MA-STP are 32° 31′ 41.4516′′ N and 44° 13′ 12.2664′′ E (Latitude: 32.528181 and Longitude: 44.220074). The treatment system used is extended aeration activated sludge (EAAS), as shown in Fig. 1. It is designed to serve 50,000 people with an estimated discharge flow rate of 8000 m^3^/day, and the operational conditions are listed in Table 1. The system consisted of aerated grit chamber with oil–water separator (API) unit, diffused aerated activated sludge unit, secondary clarifier, chlorine disinfection unit, and drying beds unit for sludge management.

**Table 1:** Operational conditions of MASTP-EAAS.

Parameter	Value
Mixed liquor suspended solids (MLSS) (mg/L)	3000
Food/microorganism (F/M) ratio (kg BOD/kg MLVSS day)	0.09
Solids loading rates (SLR) (kg MLSS/m^2^ h)	2.9
Overflow (m^3^/m^2^ day)	13
Hydraulic retention time (HRT) (h)	16
Dissolved oxygen (DO) (mg/L)	2.5
Solids retention time (SRT) (day)	25
Return activated sludge (RAS) (%)	75
Sludge volume index (SVI) (mL/g)	66
Discharge (Q) (m^3^/day)	8000
Summer temperature (°C)	27
Winter temperature (°C)	12

### Sampling and analysis

To assess the performance of the MA-STP, influent and effluent samples were monthly measured from January 2019 to December 2019. Three replicates were analyzed for pH, COD, BOD_5_, TSS, NO_3_^−^, NO_2_, NH_4_^+^, SO_4_^2−^, H_2_S, Oil & grease and PO_4_^3−^ as specified by standard procedures for analysis of water and waste water^9^, and only average values were reported. The influent and effluent characteristics is tabulated in Table 2, and the effluents were compared with the Iraqi effluent standard^10^. Throughout the year, the wind speed was ranged from 5 to 25 km/h, and the direction was mostly north-west (305°). The conversation of soluble H_2_S in wastewater to gas emitted from STP is calculated based on the following equation derived from the ideal gas law at standard conditions^11^:

1 $$ H_{2} S \;emission\; \left( {{\text{ppm}}} \right) = \frac{{\left( {V \frac{L}{{mole \;of \;H_{2} S}} } \right) \times H_{2} S\; concentration\; \left( {{\text{mg}}/{\text{L}}} \right) \;(10^{3} {\text{g}}/{\text{mg}})}}{{\left( {34.08 \frac{g}{{mole\; of\; H_{2} S}}} \right)}} $$

where V is volume occupied by the gas (L) = 22.414 L at standard temperature.

**Table 2:** Characteristics of influent in MASTP.

Parameter	Influent concentration (mg/L)	Effluent concentration (mg/L)	Permissible limits (mg/L)
pH	7.2	7	6–9
COD	450	35	100
BOD	280	10	40
TSS	300	20	60
NO_3_^−^	0	35	50
NO_2_	0	ND	–
NH_4_^+^	20	ND	10
SO_4_^2−^	1050	1175	400
H_2_S	35	1	3
Oil and grease	40	2	10
PO_4_^3−^	15	1	3

All parameters in mg/L except for pH; ND is not detected (< 1 mg/L).

### Model development

To simulate the H_2_S fate and emission from various treatment processes in MA-STP during summer and winter, TOXCHEM V4.1 simulation was used. In TOXCHEM V4.1, H_2_S has similar features like volatile organic compounds (VOC), which can be removed by liquid–gas mass transfer and biodegradation processes. In EAAS system, liquid–gas mass transfer occurs by two mechanisms: first is by volatilization to the atmosphere that is due to striping by diffused bubble aeration and volatilization from open surfaces; second is by sorption process of H_2_S to the sludge. Fate and emission processes of H_2_S in the MA-STP can be summarized in the following four methods:

1. Biological sorption of H_2_S from liquid phase to the sludge formed in the system.
2. Striping by diffused aeration that causes volatilization of H_2_S to the atmosphere.
3. Volatilization of H_2_S from open surfaces of treatment units.
4. Biodegradation of H_2_S by activated sludge process.

In the MA-STP system, air diffusers have been used to provide aeration at the grit chamber and activated sludge tank. For diffused bubble aeration, the rate of stripping is represented by concentration of pollutants in the wastewater and is written as:

2 $$ r_{d} = { }k_{d} {\text{ C }}f_{non} {\text{ V}} $$

where r_d_ is diffused aeration stripping rate (mg/h), k_d_ is diffused aeration stripping constant, C is volatile compound concentration in the water (mg/m^3^), f_non_ is pH dependent fraction of non-dissociated compound, and V is aeration basin volume (m^3^).

It is assumed that the motion of air on the top of basin (i.e. open system) is adequate to volatilize H_2_S, thus the volatilization rate is given by:

3 $$ r_{v} = { }k_{v} {\text{ C }}f_{non} {\text{ V}} $$

where r_v_ is rate of volatilization (mg/h), and k_v_ is volatilization rate constant (1/h).

Due to that MA-STP system is based on suspended growth mechanisms, suspended growth biodegradation was used in the model. Subsequently, H_2_S biodegradation is expressed by Monod reaction as shown in the following equation:

4 $$ r_{b} = k_{b} \left( {\frac{C}{{1 + \frac{C}{{K_{s} }}}}} \right)X V $$

where r_b_ is the biodegradation rate (mg/h), k_b_ is the coefficient of first order biodegradation rate (L/mg VSS/h), X is the biomass concentration (mg/L), and K_s_ is the half saturation constant (mg/L).

H_2_S transfers from the liquid phase to the suspended solids and to the residual dead biomass by mean of sorption. Sorption of H_2_S onto the sludge is described by a linear isotherm in low pollutant concentrations and it is computed by the following equation:

5 $$ q = K_{p} C $$

where q is the pollutant concentration in solid phase (µg/g), and K_p_ is the coefficient of sorption partition (L/g).

### Model validation

To evaluate the characteristic of data predicted by TOXCHEM V4.1, all measured and predicted data were compared using the statistical parameters recommended by Chang and Hanna^12^, which include fractional bias (FB), geometric mean bias (MG), normalized mean square error (NMSE), geometric variance (VG), correlation coefficient (R), and fraction of predictions within a factor of two observations (FAC2). Results of measured H_2_S values discharged with effluent and emitted to atmosphere in 12 months are compared with the predicted H_2_S values by TOXCHEM V4.1. The statistical parameters used are presented in Eqs. (6)–(11):

6 $$ FB = \frac{{\left( {\overline{{C_{o} }} - \overline{{C_{p} }} } \right)}}{{0.5 \left( {\overline{{C_{o} }} + \overline{{C_{p} }} } \right)}} $$

7 $$ MG = \exp \left( {\overline{{\ln C_{o} }} - \overline{{\ln C_{p} }} } \right) $$

8 $$ NMSE = \frac{{\overline{{\left( {C_{o} - C_{p} } \right)^{2} }} }}{{\overline{{C_{o} }} \overline{{C_{p} }} }} $$

9 $$ VG = \exp \overline{{\left( {\ln C_{o} - \ln C_{p} } \right)^{2} }} $$

10 $$ R = \frac{{\overline{{\left( {C_{o} - \overline{{C_{o} }} } \right) \left( {C_{p} - \overline{{C_{p} }} } \right)}} }}{{\sigma C_{o} \sigma C_{p} }} $$

11 $$ FAC2 = \overline{{\left( {\frac{{C_{p} }}{{C_{o} }}} \right)}} $$

where *C*_*o*_ is the measured H_2_S value, *C*_*p*_ is the predicted H_2_S value, $\overline{{C_{o} }}$ is the average over measured data, $\overline{{C_{p} }}$ is the average over predicted data, and σ is the standard deviation over the dataset. The acceptable limits for these statistical parameters are shown in Table 3.

**Table 3:** Statistical parameters of data validation of H_2_S emitted to atmosphere and discharged with effluent.

Parameter	Discharged with effluent		Emitted to atmosphere		Ideal fit	Validation limits
	Measured	Predicted	Measured	Predicted		
Average	0.48	0.37	4.46	5.98		
Standard deviation	0.21	0.17	0.29	0.35		
Fractional bias (FB)	0.25		− 0.29		0	− 0.3 ≤ *FB* ≤ 0.3
Geometric mean bias (MG)	1.28		0.75		1	0.7 ≤ *MG* ≤ 1.3
Normalized mean square error (NMSE)	0.08		0.09		0	≤ 1.5
geometric variance (VG)	1.08		1.09		1	≤ 4
Correlation coefficient (R)	0.9		0.89		1	Close to 1
Factor of two observations (FAC2) (%)	0.78		1.34		1	0.5 ≤ *FAC2* ≤ 2

### Sensitivity analysis

Among many crucial processes to understand the effect of various operational parameters on the fate and emission of H_2_S is sensitivity analysis. In this investigation, sensitivity analysis was applied to comprehend the fate and emission of H_2_S by using the major influencing parameters on the treatment process of extended aeration systems, which include aeration flowrate, H_2_S loading rate (MLSS concentration in the diffused aerated activated sludge reactor), wastewater pH level, wastewater temperature and wind speed. Different aeration flowrate (2500–15,000 m^3^/h), H_2_S loading rate (5–35 mg H_2_S/g MLSS/day), wastewater pH levels (5–10), wastewater temperatures (10–30 °C), and wind speeds (5–25 km/h) were applied.

## Results and discussion

### The performance evaluation of MA-STP

Table 2 shows the performance evaluation of the MA-STP Al-Hindiya District, Karbala, Iraq. Removal efficiency of 92, 96, 93, 100, 98, 95 and 93% were achieved for COD, BOD_5_, TSS, NH_4_^+^, H_2_S, Oil & grease and PO_4_^3−^, respectively. According to the Iraqi standards^10^, the MA-STP performed very well to remediate all pollutants, except for SO_4_^2−^. High influent SO_4_^2−^ concentration and oxidation of H_2_S result in excess presence of SO_4_^2−^ concentration in the system^13^, therefor it is higher in effluent than the influent. However, neutral pH and high degradation of organics indicated a stable biological process. NH_4_^+^ and NO_2_ was not detected in the effluent due to complete nitrification process achieved by the EAAS system^8^. In contrast, about 35 mg/L of NO_3_^−^ was observed in the effluent because the treatment system does not include denitrification process that need to be considered to improve the system performance. Furthermore, high oil & grease removal attributed to the application of oil–water separator (API) in the aerated grit chamber. Besides, dissolved H_2_S was detected at trace level in the effluent, because most of it was degraded in the treatment process and the rest was emitted to the atmosphere.

### Results validation

TOXCHEM V4.1 model is used to simulate the H_2_S fate throughout the MA-STP and emission out of it. The influent characteristics and EAAS system operational conditions were the inputs, and H_2_S fate (% and mg/L) and emission values (ppm) were the output of the model. From these applied characteristic and operational variables, model simulations were generated and compared with H_2_S analysis in the sampling points of emitted H_2_S at the top of each treatment unit and dissolved H_2_S with effluent. Table 3 presents statistical data validation of predicted and measured H_2_S emitted to atmosphere and discharged with effluent. In comparison, all data were validated within the limits, and dataset NMSE, VG, and R were close to the ideal fit.

FB and MG measure mean bias and indicate systematic errors which lead to underestimate or overestimate the measured data. FB of 0.25 (more than zero) and MG of 1.28 (more than one) evidence that TOXCHEM under predicted H_2_S concentration discharged with effluent, while FB of − 0.29 (less than zero) and MG of 0.75 (less than one) indicate that the model over predicted H_2_S emission to atmosphere. However, both of FB and MG showed that the error in all data are within acceptable limits and less than 30%. NMSE and VG showed that data scattering around the true value and they both reflected systematic random errors from unpredictable fluctuations. The results of both of NMSE and VG are very close to ideal fit, indicating that there is no random error for the predicted data over measured.

The coefficient of correlation (R) reflects the linear relationship between modeled and observed data. Both R values, 0.9 for H_2_S concentration discharged with effluent and 0.89 for H_2_S emission to atmosphere, indicated a strong correlation between predicted and measured data. The highest R values is required but not sufficient, therefore FAC2 is important factor for evaluation and validation as it’s the most robust measure that is not affected by either low or high outliers. The results of FAC2 revealed that 78% (FAC2 = 0.78) of H_2_S concentration discharged with effluent and 75% (FAC2 = 1.34) of H_2_S emission to atmosphere were within a factor of two of the measured data.

**Figure 2:**
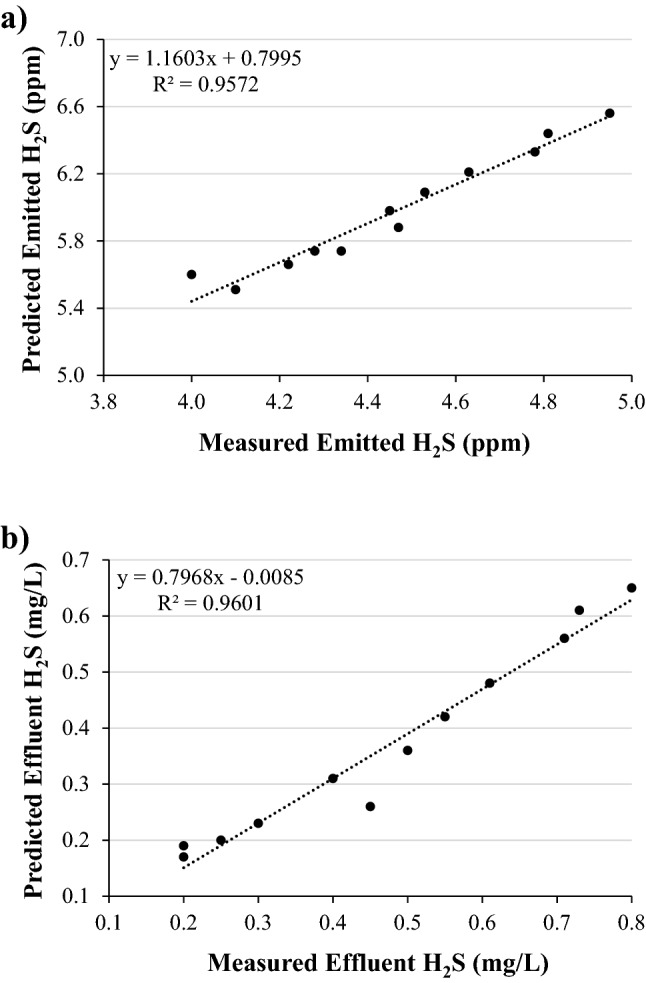
The comparison of measured (x-axis) and predicted (y-axis) H_2_S: (**a**) emitted to atmosphere and (**b**) discharged with effluent.

In addition, Fig. 2 shows a scattering comparison of measured and predicted H_2_S that are emitted to atmosphere and discharged with effluent. Distribution of data and coefficient of determination (R^2^) are adopted to check the goodness of model fit. The results showed that the predicted and measured data were well scattered around the linear line, where measured emission was slightly less than predicted and measured discharged concentration was slightly higher than the predicted, and R^2^ values showed that data are in a good fit. The TOXCHEM model could sufficiently describe the experimental data of H_2_S fate and emission. In comparison with other studies on the modeling of H_2_S using AERMOD^7^, CALPUFF^14^, and GOSTELOW^15^, statistical analysis of TOXCHEM V4.1 model are very satisfactory to study H_2_S due to valid prediction with less limitations and errors.

### H_2_S fate and emission

**Figure 3:**
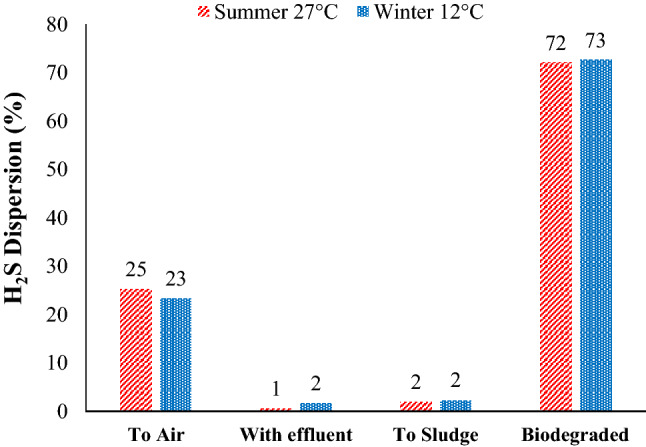
H_2_S dispersion (%) throughout the EEAS system in MA-STP during summer and winter.

STP are a major source of gaseous emissions that contain odorants and greenhouse gases. Figure 3 shows the H_2_S fate (%) throughout the MA-STP. The EAAS system receives about 280 kg/day of H_2_S that is processed throughout the treatment units. During summer (27 °C) and winter (12 °C), about 25 and 23%, 1 and 2%, 2 and 2%, and 72 and 73% were fated as emitted to air, discharged with effluent, sorbed to sludge, and biodegraded, respectively. The results revealed that the major processes occurring are: (1) degradation, where most of the H_2_S was oxidized by aerobic process; and (2) emission, where some of the H_2_S was emitted to the atmosphere by H_2_S stripping and vitalization from open surfaces. In addition to seasonal variation, sorption of H_2_S to the dead biomass and discharge of H_2_S with effluent were slightly observed. Although the key function of activated sludge process is to eliminate organic pollutants, the EAAS system has successfully achieved desulfurization of about 74% of H_2_S (degradation and sorption).

In an aerobic conditions, natural microorganisms called sulfide oxidizing bacteria (SOB) play a major role in the desulfurization of H_2_S. H_2_S is oxidized by chemolithoautotrophic bacteria from the genus *Thiobacillus* group that has high affinity to sulfide compounds (H_2_S, HS^−^ and S^2−^)^16^. In aqueous solution, H_2_S presents in forms that are highly depending on pH level. As the sewage pH is about 7 at STP, H_2_S is primarily dissociates to form bisulfide (HS^−^) (Eq. (12)). Sulfide (S^2−^) is another form of H_2_S (Eq. (13)) that is generally neglected because of its insignificant presence except at very high pH, and H_2_S form may be predominant below pH 5^13^.

12 $$ {\text{H}}_{{2}} {\text{S}}_{{({\text{aqueous}})}} \to {\text{ HS}}^{ - } + {\text{ H}}^{ + } \left( {{\text{pKa }} = { 7}.0{5},{ 5 } < {\text{ pH }} \le { 1}0} \right) $$

13 $$ {\text{HS}}^{ - } \to {\text{ S}}^{{{2} - }} + {\text{ H}}^{ + } \left( {{\text{pKa }} = { 12}.{9},{\text{ pH }} \ge { 11}} \right) $$

In STP where aeration is provided, HS^−^ is biologically oxidized to firstly elemental sulfur (S^0^) and subsequently to sulfate (SO_4_^2−^), as shown in the following reactions^17^:

14 $$ {\text{HS}}^{ - } + \, 0.{\text{5 O}}_{{2}} \to {\text{ S}}^{0} + {\text{ OH}}^{ - } $$

15 $$ {\text{S}}^{0} + {\text{ OH}}^{ - } + { 1}.{\text{5 O}}_{{2}} \to {\text{ SO}}_{{4}}^{{{2} - }} + {\text{ H}}^{ + } $$

Complete oxidation of HS^−^ to SO_4_^2−^ requires the consumption of two oxygen molecules, but this reaction is reversible if limited amount of oxygen is supplied and elemental S^0^ might accumulate^13^. However, elemental S^0^ is end-product of oxidation process that is necessary for the growth of microorganisms and directly consumed for the synthesis of cellular protein needed for new cells production^18^. Excess amounts of elemental S^0^ and SO_4_^2−^ are sorbed to the biomass and/or released with the effluent.

**Figure 4:**
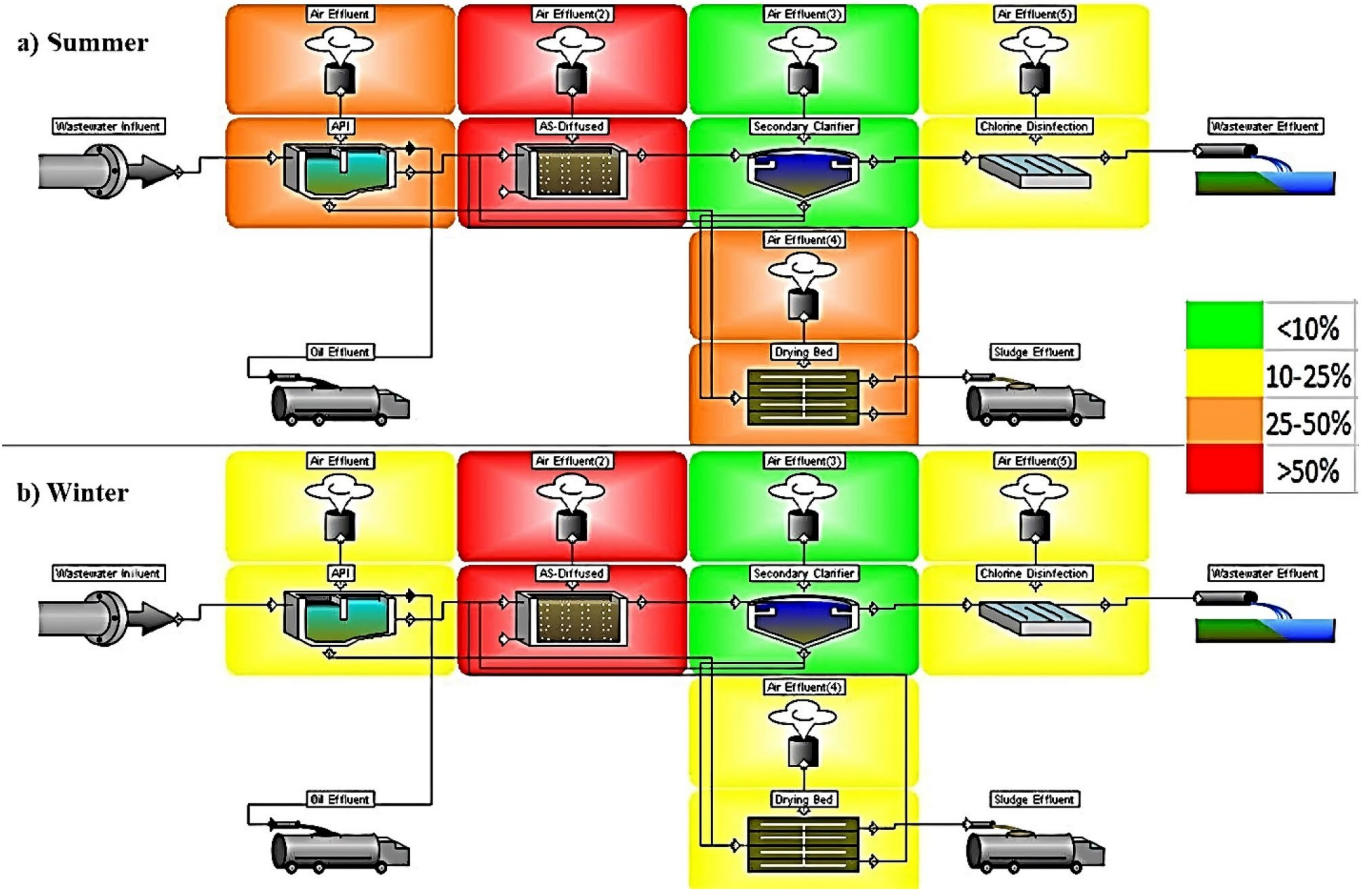
Emission of H_2_S to atmosphere from each unit (% of the total emission): (**a**) summer and (**b**) winter.

Volatilization describes the process whereby an odorant (H_2_S) is transferred from an area source such as the surface of diffused aerated activated sludge reactor to the atmosphere^15^. Figure 3 displays that about 70 kg/day (23%) of total H_2_S was volatized from the MA-STP to atmosphere, and Fig. 4 shows the emission distribution of H_2_S from each unit (% of the total emission). The results revealed that summer has emitted higher H_2_S compared to winter, in which most of it was from diffused aerated activated sludge reactor (> 50%), followed by aerated grit chamber (API) (25–50%) and sludge drying beds (25–50%). The mechanism of H_2_S emission is volatilization by air stripping and open surfaces. The H_2_S emission is a physicochemical process that contains liquid and gas phases. Only H_2_S (aqueous) can transfer across the sewage-air interface, allowing it to be emitted as gas from STP^19^, as shown in Eq. (16).

16 $$ {\text{H}}_{{2}} {\text{S}}_{{({\text{aqueous}})}} \to {\text{ H}}_{{2}} {\text{S}}_{{({\text{gas}})}} \left( {{\text{K}}_{{\text{C}}} \approx {\text{ 468 atm}}/{\text{mole fraction}}} \right) $$

A STP brings huge quantities of sewage into contact with air that boost the stripping of odorants, which can be significant odor source. Due to aeration process, biological oxidation tends to decrease liquid-phase odorant concentrations. However, recycling of activated sludge is a notable way for odor control due to the recycling of biomass containing sulfur compounds from secondary clarifiers to the aerobic activated sludge reactors. This fosters the consumption of odor compounds before they volatilize from the liquid phase to the atmosphere^16^. Furthermore, the available surface area for gas transfer is believed to affect the emission of H_2_S from open surfaces to the atmosphere. This was also proved by Parsons et al*.*^20^ whom found that the greater open surface area of the source the greater H_2_S concentration emitted to atmosphere.

**Figure 5:**
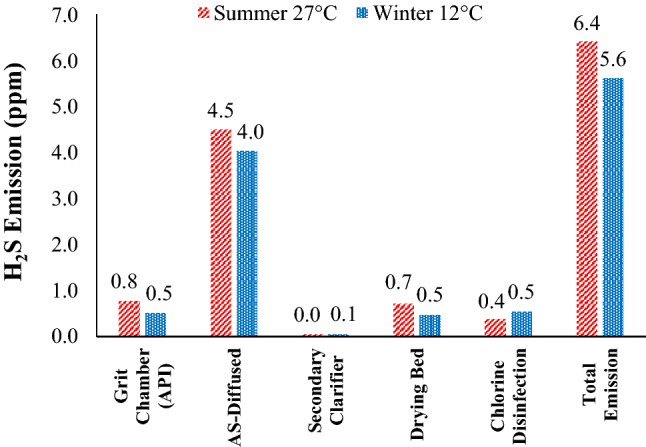
Emission of H_2_S to atmosphere from each unit (ppm) during summer and winter.

Figure 5 shows the concentration of H_2_S (ppm) emitted to atmosphere from each unit. At summer and winter, the total emission were 6.403 and 5.614 ppm, from diffused aerated activated sludge reactor were 4.492 and 4.035 ppm, from aerated grit chamber (API) were 0.768 and 0.507 ppm, from sludge drying beds were 0.718 and 0.475 ppm, from secondary clarifier 0.379 and 0.541 ppm, and from chlorine disinfection were 0.046 and 0.056 ppm, respectively. The results indicated that H_2_S emission from all units was within the human odor threshold (0.0005–1.5 ppm)^3^, except for diffused aerated activated sludge reactor that was much higher. Long human’s exposure (8 h) to concentrations higher than 5 ppm (total emission in this study) may cause headache, nausea and tearing of eyes. Therefore, MA-STP workers are exposed to health rick due to their exposure to high concentrations of H_2_S that required odor control system (especially at diffused aerated activated sludge reactor), the modification of operational process, or/and shorter working schedule.

### Sensitivity analysis

**Figure 6:**
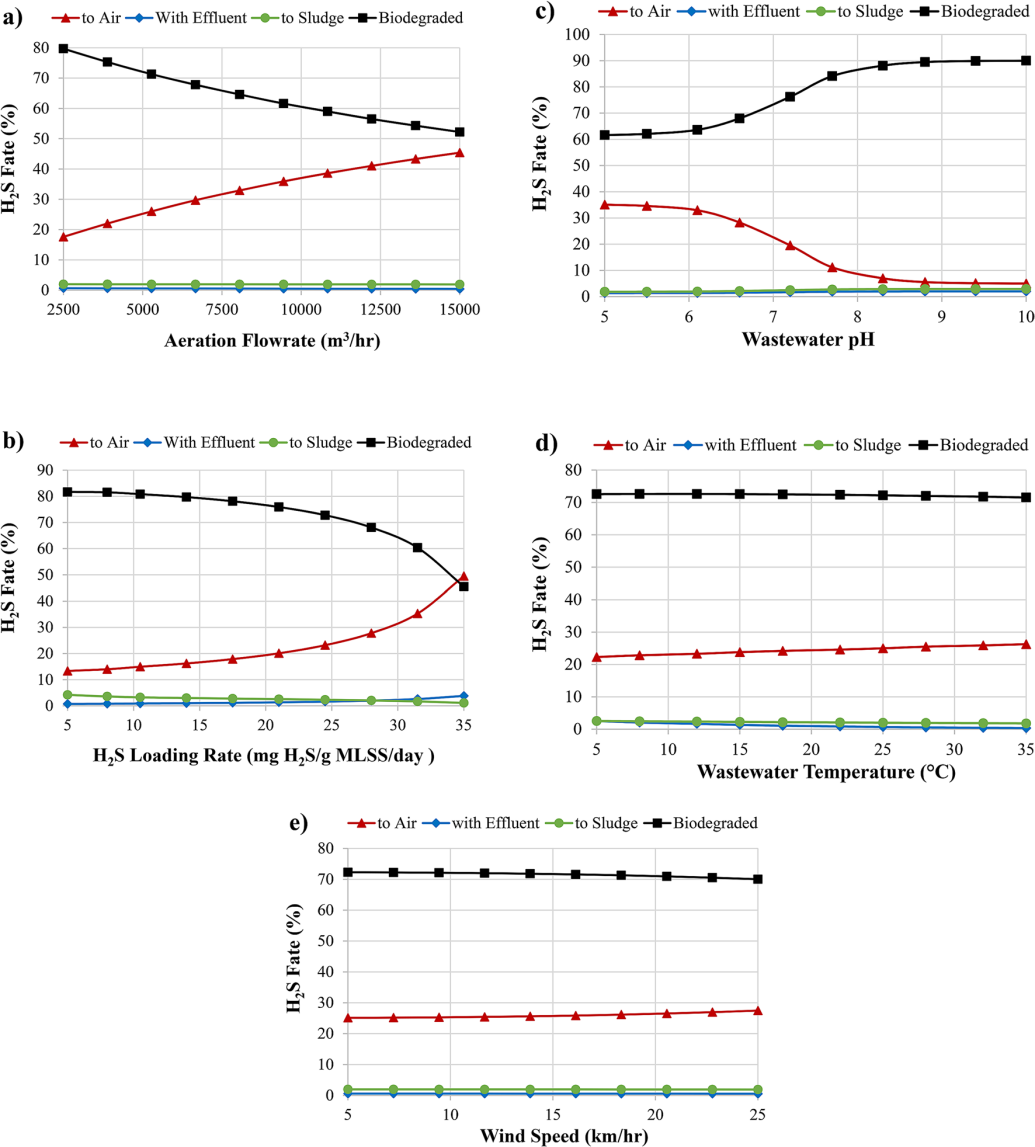
Sensitivity analysis of different H_2_S dispersion: (**a**) aeration flowrate, (**b**) MLSS concentration, (**c**) wastewater pH level, (**d**) wastewater temperature, and (**e**) wind speed.

H_2_S fate and emission within/from EAAS system are affected by operational parameters such as aeration flowrate, H_2_S loading rate, wastewater pH level, wastewater temperature and wind speed. Figure 6a demonstrates the effect of aeration flowrate (2500–15,000 m^3^/h) on the fate of H_2_S. H_2_S sorption to sludge and discharge with effluent was not affected by change in aeration flowrate, compared to biodegradation and volatilization processes. Interestingly, increase in aeration flowrate from 2500 to 15,000 m^3^/h has increased the emission of H_2_S to atmosphere from 18 to 45%, and decreased biodegradation process from 80 to 52%. The authors cannot negate that there are several evidences on aeration causes odorants stripping by air bubbles, for instance, Baawain et al*.*^2^ reported that emissions of H_2_S was intensified by air bubbles during the aeration process. In another study by Tzvi and Paz^13^, they stated that 15–30% of H_2_S was evaporated within the air bubbles introduced to the system and released to the atmosphere, which was much higher than operating the system in absence of bubbles streams. In aeration reactor, H_2_S emission to the atmosphere by stripping and volatilization from open surfaces may occur first, then followed by oxidation of H_2_S by aerobic microorganisms. Hence, monitoring H_2_S emission from aeration stream is not only necessary to evaluate H_2_S fate but also for safety aspects. Therefore, operating the EAAS system at lowest aeration flowrate will reduce the emission of odorants and increase biodegradation treatment.

Figure 6b describes the effect of H_2_S loading rate (MLSS concentration in the diffused aerated activated sludge reactor) on the fate of H_2_S. It is notable that decrease in the H_2_S loading rate (increase in MLSS concentration) from 35 to 5 mg H_2_S/g MLSS/day has enhanced biodegradation process from 45 to 82%, improved sorption process from 1 to 4%, decreased emission to atmosphere from 50 to 13%, and reduced discharge with effluent from 4 to 1%. There is an inverse correlation between H_2_S loading rate and removal efficiency^21^, in which an increase in H_2_S loading rate will first decrease the biomass activity resulting in lower biodegradation process leading to decreased H_2_S removal efficiency, and second increase aqueous H_2_S concentration available for H_2_S emission to atmosphere and/or discharged with effluent.

Figure 6c displays the effect of wastewater pH level on the fate of H_2_S, in which it effects first the dissociation of H_2_S in aqueous solution, and second the mechanism of H_2_S removal. The results vouch that increase in pH between 5 and 10 decreased the emission of H_2_S to atmosphere from 35 to 5%, making it more dissolved in sewage and increased biodegradation process from 62 to 90%, whereas sorption to sludge and discharge with effluent were not much effected. As pH increases, the fraction of available H_2_S decreases due to its dissociation into HS^−^ (Eqs. (12), (13)). Higher pH solution led to less H_2_S available for transferring from STP treatment units into the atmosphere, whereas H_2_S stripping is favored under acidic conditions^17^. Chaiprapat et al*.*^22^ observed that as pH of the wastewater decreased, the efficiency of H_2_S removal of the system slightly decreased due to lowered solubility of H_2_S, which lead to higher ionic strength of wastewater. Low solubility makes H_2_S and O_2_ in gas form become deficient for SOB to execute biochemical reactions in the liquid form.

Moreover, the oxidation of sulfide compounds produces H^+^ (Eq. (17)), leading to drop in the pH in the aerobic unit of STP. Low pH level may inhibit biodegradation process because under acidic environment, H_2_S is unionized and has neutral molecule that is very toxic to microorganism in the system as it can permeate through the cell membrane better than HS^–^ and S^2–^^1^. However, continuous wastewater feeding in STP provides recirculation and alkalinity buffering to maintain pH and hinder acidity. This indicate that using aeration for biological oxidation wouldn’t results in external release of H_2_S, and decrease the risk of H_2_S stripping.

17 $$ {\text{HS}}^{ - } + {\text{ 2O}}_{{2}} \to {\text{ SO}}_{{4}}^{{{2} - }} + {\text{ H}}^{ + } $$

Temperature is another key factor influencing the physicochemical properties of gases, influencing the Henry gas law and kinetics of biological processes. Figure 6d presents the effect of wastewater temperature ranges from 5 to 35 °C on the fate of H_2_S. It was observed that temperature mainly effected the mass transfer of H_2_S, either dissolved in wastewater or volatized to atmosphere. Increase in temperature from 5 to 35 has increased the emission of H_2_S from 22 to 27% and decreased its content in wastewater from 3 to 0%, while effects on degradation and sorption processes were limited. The findings evince that aqueous H_2_S condensed at a lower temperature and emitted to atmosphere at high temperature. Similarly, Baawain et al*.*^2^ confirmed that high temperature has increased H_2_S emissions from ponds sewage treatment system. Other studies also reported that aqueous solution temperature highly effected the mass transfer rate of H_2_S, in which the overall mass transfer from liquid phase to gas phase increases with temperature^6^.

Figure 6e display the effect of wind speed (friction velocity) on the emission of H_2_S from STP to the atmosphere. It was seen that increase in wind speed from 5 to 35 km/h has slightly increased the volatilization of H_2_S from 25 to 27% and decreased degradation process from 72 to 70%, whereas sorption and dissolution of gas in wastewater processes were not affected. However, this can show that wind speed has limited effect on the gas emission but of course will highly influence the dispersion of odors away from its generation source. Similarly, slight higher emission rate of H_2_S was observed with higher wind speed^2^. Wind speed is usually correlated with mass transfer and emission, where it is evident on wind speeds over 4 m/s, and nearly undetectable below this speed^5^. However, the wind speed reported to be associated with H_2_S concentration more than the emission rate, in which higher wind speed dilutes the concentration of H_2_S and disperses it for long distance^6^. In this regards, Santos et al.^15^ reported that wind speed did not have a significant effect on overall mass transfer of H_2_S, suggesting that volatilization will depend more on turbulence of liquid phase than wind speed.

## Conclusion

TOXCHEM V4.1 simulation showed that EAAS system worked as biological treatment method for the removal of H_2_S. The main processes occurring in the EAAS system are (1) H_2_S compounds (HS^−^) formation, (2) H_2_S biological degradation, (3) H_2_S volatilization, (4) H_2_S stripping, (5) H_2_S compounds sorption, and (6) discharged H_2_S with effluents. The date predicted by TOXCHEM V4.1 simulation were validated and close to ideal fit. The main H_2_S processes observed were degradation by about 73% and stripping by about 23%. Total H_2_S emission from the MA-STP, especially from diffused aerated activated sludge reactor, may put the workers and surrounding population at a health risk. Operating the EAAS system at low aeration flowrate, high MLSS concentration, and slightly high pH are recommended to limit the emission of H_2_S to the atmosphere. Thus, TOXCHEM V4.1 model can potentially be utilized for other plants/projects to predict H_2_S fate and dispersion, and analysis of its results can be used as a beneficial output for decision makers.
